# Matrimonial

**Published:** 1856-06

**Authors:** 


**MATRIMONIAL.**

Married—In Dayton, Ohio, early in May last, Professor S. G. Armor, of Cincinnati, to Miss Holcomb, of the former place.

Prof. Armor was for some time our worthy colleague, and during his connexion with the College at this place, he, with two others constituted a *trio* of friends. One of his *chums* was a sound lawyer, a man of superior intellect, and of undoubted purity of character. Blackstone, Chitty, and other great legal authors had levied a claim upon his attention and affections, and so completely engrossed them, that it was supposed that nothing could reach his feelings, or claim his attention, unless it was bound in "calf" and was a treatise upon the *lex justitia.* But in a moment of relaxation, a pair of sparkling black eyes and a beautiful bright-smiling face constituted a page which awakened new thoughts and new emotions. His fate from this hour was sealed, and he surrendered himself to the power of the band of Benedicts. The other a shrewd financier as well as a very intelligent man and a gentleman, was then and is yet, the companion of piles of gold and silver coin upon his bank table, and such his fondness for bills, draughts, acceptancies, and securities, that his career has been onward and never "checked" by any object. Devoted to his gains, in which he is not only eminently, but deservedly successful, and married to his pursuit, he is so much a puritan that he will not perpetrate a moral polygamy. He resists the power of the captivating smiles and contemns Benedicts. In this he was fully sustained by the example of his friend and fellow *chum*, the Dr.

But now he stands alone, for the latter has "gone the way" of all those who have been wounded by the arrow of Cupid, and turning from the study of life, health and disease, he threw down his books, charts, and specimens, then doffing his professional robes, he vowed most complacently to one who had overcome, by some means, his proclivities and partialities for "old Bachelordom," and upon the most solemn promises to do better in the future, he was forgiven the past. We had recently the satisfaction of seeing him "regenerated and disinthralled" and "the happiest of the happy" and we thought in truth, he had good reason to be, *Requiesent in pace.*

The remaining member of the "*trio*" upon learning the particulars of this last casualty," of which we have given notice above, felt himself abandoned, and in order to dissipate the loneliness of his condition, has taken to wandering. We would not be surprised if he met with an object, during his absence, which would withdraw his attention, for the time being at least, from the contemplation of the *cent per centum*, to which we would say, Amen! So mote it be.

